# Smartphone-Based Traveled Distance Estimation Using Individual Walking Patterns for Indoor Localization

**DOI:** 10.3390/s18093149

**Published:** 2018-09-18

**Authors:** Jiheon Kang, Joonbeom Lee, Doo-Seop Eom

**Affiliations:** Department of Electrical Engineering, Korea University, Seoul 02841, Korea; kanghead@korea.ac.kr (J.K.); sldofvge12@korea.ac.kr (J.L.)

**Keywords:** indoor localization, smartphone-based pedestrian dead reckoning, stride length estimation, time-series signal deep learning framework

## Abstract

We introduce a novel method for indoor localization with the user’s own smartphone by learning personalized walking patterns outdoors. Most smartphone and pedestrian dead reckoning (PDR)-based indoor localization studies have used an operation between step count and stride length to estimate the distance traveled via generalized formulas based on the manually designed features of the measured sensory signal. In contrast, we have applied a different approach to learn the velocity of the pedestrian by using a segmented signal frame with our proposed hybrid multiscale convolutional and recurrent neural network model, and we estimate the distance traveled by computing the velocity and the moved time. We measured the inertial sensor and global position service (GPS) position at a synchronized time while walking outdoors with a reliable GPS fix, and we assigned the velocity as a label obtained from the displacement between the current position and a prior position to the corresponding signal frame. Our proposed real-time and automatic dataset construction method dramatically reduces the cost and significantly increases the efficiency of constructing a dataset. Moreover, our proposed deep learning model can be naturally applied to all kinds of time-series sensory signal processing. The performance was evaluated on an Android application (app) that exported the trained model and parameters. Our proposed method achieved a distance error of <2.4% and >1.5% on indoor experiments.

## 1. Introduction

Recently, the proportion of time in daily life spent in indoor spaces, such as the home, office, and shopping mall complexes has gradually increased, and the services have been normally provided outdoors, such as car navigation systems, are gradually expanding to provide indoor positioning or navigation systems. Although indoor positioning is difficult to conduct using the global position service (GPS) because GPS signals cannot be received, technical studies are now being conducted to provide various indoor location-based services [1]. In particular, smartphones with various sensors such as accelerometers, gyroscopes, magnetometers, and barometers have become popular, and network infrastructures (e.g., Wi-Fi) have expanded to a point at which location-based services are achievable for indoor spaces [2].

Indoor localization techniques can be classified into various categories according to the required location accuracy, available service area, specific application, and available sensors, and these can be implemented using propagation model-based, fingerprint, and dead reckoning (DR) methods [2]. A propagation model method estimates the user’s location based on the received signal strength (RSS) according to radio propagation, and the performance can vary according to the distortion of the radio waves and the placement density of the reference points (e.g., access points and beacons) in an indoor environment [3]. A fingerprinting method builds a feature map through a pre-collection step at pre-defined points of the indoor map, and it compares the measured signal to the collected feature map, and then determines the position on the indoor map with a high similarity [4]. However, there are disadvantages in that the collection cost increases due to the pre-collection step, and the correction and recollection process of the data map are required in the case in which the indoor environment changes [5]. A dead reckoning method estimates the current position based on the distance moved and the direction using an inertial measurement unit (IMU). Even then, a low accuracy of the low-cost Micro Electro Mechanical System (MEMS) sensor causes positioning error accumulation, and a DR can independently make an estimate without additional infrastructure and does not require a pre-collection process [6].

In general, pedestrian dead reckoning (PDR) uses an IMU to determine the current user location by estimating the step count, stride length, and direction [7]. In the case in which the IMU is attached to the ankle, the zero velocity update (ZUPT) and the zero angular rate update (ZARU) method based on the walking model can be used to estimate the number of steps, stride length, direction, and can compensate for the drift error that occurs in the IMU sensor [8]. However, it is difficult to reflect the walking models or patterns (e.g., holding a device in hand, swinging, and keeping in a pocket or bag) of a pedestrian using the PDR method with a smartphone [9]. In addition, most of the literature suggests a generalized formula that reflects a feature of the IMU signal from several subjects in a given experimental walking and running scenario [10,11,12].

**Figure 1:**
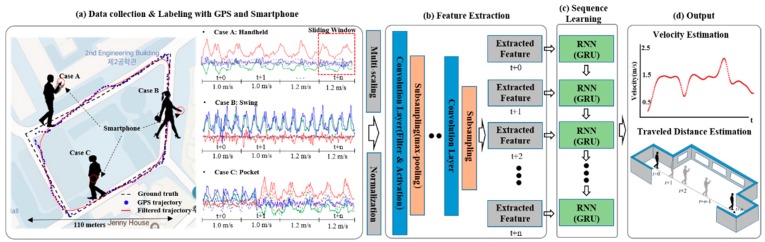
An overview of our indoor localization system using a proposed outdoor walking pattern learning scheme. (**a**) Inertial measurement unit (IMU) signal and velocity mapping using global position service (GPS)-based user trajectory by walking pattern at outdoor. The collected dataset is used as training data to the following proposed deep learning model; (**b**) Hybrid CNN–RNN deep learning model. The nonlinear features of the training data were automatically extracted using the multiple convolutional layers with activation and pooling layers; (**c**) The RNN uses the extracted feature vector at each time step as an input, and learns using signal patterns and labeled velocities; (**d**) The pedestrian’s average velocity at each time step and the cumulative traveled distance using the learned deep learning model parameters are estimated when moved indoors.

In contrast with other studies, our method does not require any manually-designed features, and we explore a learning-based approach for indoor localization. Figure 1 shows an overview of our proposed system, which is composed of three phases. The first phase involves data collection and labeling. The main motivation of our study was to estimate the traveled distance regardless of the pedestrian’s walking type, smartphone orientation, position, and shaking. In order to assign a constant label to variable sensor signal patterns in the above condition, we applied the average velocity over the unit time (e.g., 1 s) based on the corrected GPS trajectory. In other words, the velocity of the pedestrian is calculated in the GPS available area, and the dataset is constructed by mapping the velocity as a label and the IMU sensory signal as data generated at the same time step. A hybrid convolutional–recurrent neural network (CNN-RNN)-based supervised learning phase is next. The sensory inputs are transformed into nonlinear feature vectors that are utilized as the input of recurrent neural networks (RNNs), such as long–short term memory (LSTM) and gated recurrent unit (GRU) by multiple convolutional and subsampling layers automatically. The GRU [13] model learns between the input feature vector and the velocity label considering temporal sequence information recurrently. Finally, based on the learned deep learning model parameters, the raw sensory signal is estimated as the average velocity and cumulative traveled distance per time step when moving indoors.

To summarize, we proposed our own walking pattern using an outdoor learning method based on a deep learning and indoor localization method using the learned parameters. This study focuses on estimating the distance traveled, which is one of the key elements of the DR method. The contributions of our study are summarized below:

(1) *Outdoor walking patterns are learned and then applied to indoor localization.* To learn the individual user’s walking patterns, the trajectory with a GPS error was corrected for the user, and along with the IMU sensor signal was mapped on the available GPS area using the user’s own smartphone. Indeed, our proposed approach is more effective in terms of the device, user, and walking pattern diversities compared to conventional manually-designed feature extraction.
(2) *Estimation of the average moving speed for segmented IMU sensor signal frames.* In the case of conventional PDR, the traveled distance is estimated by calculating the step count and stride length using the handcrafted features of the IMU signal. However, we proposed a scheme to estimate the traveled distance by calculating the average moving speed and duration of the signal frame.
(3) *Combination of multiscaling for automatic pre-processing at different time scales and CNNs for nonlinear feature extraction and RNNs for temporal information along the walking patterns*. Multi-scaling makes the overall trend for different time series input signals. Several stacked convolutional operations create feature vectors from the input signal automatically, and a recurrent neural network model deals with the sequence problems.
(4) *End-to-end time series classification model without any handcraft feature extractions as well as requiring any signal or application specific analysis*. Many of the existing methods are time-consuming and labor-intensive for feature extraction and classification, and these are limited in their domain-specific application. However, our proposed framework is a general-purpose approach, and it can be easily applied to more kinds of time-series signal classification, regression, and forecasting.

The remainder of the paper is organized as follows. Section 2 provides the related works and motivation of this work. Section 3 describes the proposed methods for personalized pedestrian step length estimation for indoor localization using a smartphone. Section 4 then summarizes the performance of our proposed model to produce the results of indoor localization. Finally, Section 5 summarizes and concludes our work.

## 2. Related Works

### 2.1. Indoor Localization

There are a number of existing methods for indoor localization, including radio frequency, magnetic field signal, IMU sensors, and hybrid approaches [2]. Smartphone-based methods can employ any type of indoor localization techniques using built-in sensors (e.g., accelerometers, gyroscopes, magnetometers, camera, wireless local area network (WLAN) and bluetooth low energy (BLE)), except when additional infrastructure and hardware components are required, such as ultra-wideband (UWB) [14] and WiFi channel state information (CSI) [15]. Therefore, we describe the need and motivation of our study through existing literature. 

Indoor localization using smartphones has been widely studied based on wireless signals as a mainstream approach, since most buildings have WLAN access points (AP) and smartphones have WLAN connectivity, and BLE is also available. Using received signal strength (RSS) measurements, the user location can be estimated using multilateration converted to distance from a smartphone to several reference points [16] or a comparison with a pre-collected radio map [17]. However, the accuracy of the RSS-based approach can be affected by the device orientation, attenuation due to human bodies or walls, the density of reference points, and changes in the indoor environment (e.g., removed or added reference points).

Using the distortion in the Earth’s magnetic field caused by a metal structure in an indoor environment, a constant signal map can be constructed at a fingerprinting point rather than radio frequency signal-based approaches [18]. In general, the fingerprinting approach makes a database of magnetic field distributions through offline collection and mapping, and then compares the measured signal at arbitrary points with the database to find a corresponding location [19]. Therefore, the accuracy depends significantly on the resolution of the measurement points. Even though additional infrastructure is not required, such as APs and beacons, the collection and maintenance of high quality signal mapping is a time-consuming and labor-intensive process.

The major advantage of a PDR approach is that it is possible to estimate as a standalone, and it does not require additional infrastructure or any pre-collection of a database, because the current position of the pedestrian is estimated according to the moving distance and direction from the previous position using an IMU sensor. Conventional PDR methods consist of three phases: (i) step counting, (ii) step length estimation, and (iii) walking direction estimation [20]. The number of steps is determined by the threshold, peak detection, or zero-crossing method in a periodic change of the accelerometer signal [21,22]. The step length is computed using the generalized formulas related to the magnitude of the accelerometers and gyroscopes, frequency of the step, and height of the pedestrian [11,12,23]. The walking direction estimation uses gyroscopes and magnetometers to detect the relative direction for a short time and a smartphone’s orientation in a global frame [24]. Considering the low accuracy of the MEMS sensor, the accumulated sensor error, and various pedestrian movements, indoor positioning is still a challenging problem when using smartphones particularly.

Even though each of the technical approaches described above has its strong and weak points, there is no single method that can be applied for all indoor spaces and services. Naturally, some of the literature indicated that two or more techniques have been blended. A typical combination involves Wi-Fi or BLE with PDR, and this can improve the accuracy by calculating the global absolute position that is less accurate using network infrastructure and combining the PDR with a locally higher relative position [2,9]. In Bao et al. [25], map matching is presented as a localization solution. To compensate for the accumulating and drifting error in the PDR, the map environment information, such as the distance of the corridor and the position of corner, was used (e.g., the length of the corridor is utilized to calibrate the average step length).

Although complementary techniques and calibration methods can increase the accuracy, individual localization technologies must be improved in order for the indoor localization system to be applied in a manner that is more precise and applicable to real life. Therefore, we proposed a deep learning-based traveled distance estimation method, even if the movement of the pedestrian and the placement of the smartphone change dynamically.

### 2.2. Deep Learning for Time-Series Sensory Signal Analysis

Deep learning is part of machine learning based on a set of algorithms that attempt to model high-level abstractions in data by using multiple nonlinear transformations. Recently, deep learning has become a state-of-the-art machine learning method, such as image classification, video recognition, and natural language processing [26]. In particular, CNN and RNN are excellent for automatic nonlinear feature extraction and the exhibition of temporal behavior for a time sequence on their input, respectably [27]. Many studies have already presented remarkable performance through CNN and RNN as well as a hybrid model compared to traditional signal processing based on handcraft feature extraction in time-series signal analysis.

In Kiranyaz et al. [28], a CNN model was applied in an anomaly detection with a one-dimensional (1D) electrocardiogram signal. Similarly, the goal of the approach in Ince et al. [29] and Abdeljaber et al. [30] was to autonomously learn useful features to monitor the mechanical condition, such as the motor and bearing fault based on a raw 1D signal. CNN and RNN models are widely used in human activity recognition using multiple sensor signals from smartphones as well [31,32]. In addition, DeepSense integrated CNN and RNN automatically extracts features and relationships on local, global, and temporal cases, and their experiments have shown that the feature vectors extracted by CNN can be effective when fed into RNN as inputs [33].

Deep learning for PDR-based indoor localization has been used to count the number of steps and estimate the stride length. Edel et al. [34] proposed a step recognition method based on BLSTM (bidirectional long-short term memory)-RNNs with three-axis acceleration from a smartphone. In Xing et al. [35], five manually-designed parameters from the IMU signal that are attached to the top of the foot and closely related to the linear walking model are fed into the artificial neural network (ANN) as input, and the desired network output is the stride length obtained through a regression. Hannick et al. [36,37] proposed a CNN-based stride length estimation and gait parameter extraction methods using publicly available datasets collected through an IMU attached to the shoe.

Recently, the number and type of sensors used in the internet of things (IoT), cyber physical systems (CPS), and wireless sensor networks (WSN) have been increasing, and also the field to utilize sensor data has become wider. In such a situation, deep learning is a good tool to exploit and analyze collected data without the need for special domain knowledge or signal processing techniques. Therefore, we considered a general time-series sensory signal classification framework that accommodates various fields of application, and reflects our proposed model, which referred to multiscale hybrid convolutional and recurrent neural network design. However, the deep learning approach is not always straightforward when calculating an optimal result compared to manually designed processing architectures.

## 3. The Proposed System Design

The main contribution of our study is to learn the outdoor walking pattern and then use it in an indoor environment. To enable this approach, the moving speed is calculated by the corrected user trajectory, and an appropriate deep learning model should be designed for feature extraction and training of the measured IMU sensory signal with labeled speed.

### 3.1. Automatic Dataset Collection Using the Corrected Pedestrian Trajectory with Kalman Filter

Our proposed method requires calculating the velocity per unit time using the corrected and filtered pedestrian trajectory based on raw GPS positioning. In most of the prior literature applying general purpose signal processing or deep learning models, the traveled distance is estimated by calculating the step count and stride length. However, we proposed a novel scheme to estimate the traveled distance by calculating the average moving speed and time duration using a deep learning approach with segmented IMU signal samples as the input.

One of most important things that can improve the performance in deep learning is the collection of large, high-quality datasets. It is quite a challenge to assign a label in real-time or automatically to a time-series signal that is measured on a densely attached sensor on the body or carried smartphone. Therefore, many studies manually assign a label to the measured sensor signal in a limited experimental condition (e.g., after a certain period of walking or running with a fixed gait, smartphone placement, orientation, and same stride, through assigned a fixed stride as a label for the entire measured signal data).

We proposed a method to automatically configure the dataset outdoors where a GPS signal can be received with a high reliability. However, since the GPS contains a positional error, we use a Kalman filter to correct it. The Kalman filter is a method to estimate the optimum and filtering error by removing the statistical noise included in a series of measurements observed over time.

**Figure 2:**
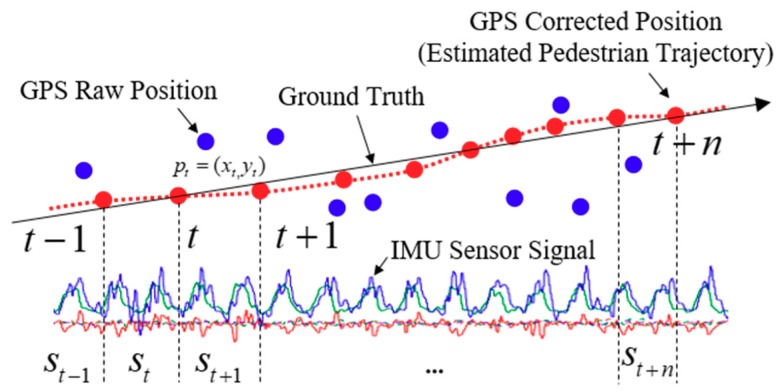
The proposed dataset collection scheme for pedestrian walking patterns in real-time, automatically. The blue dots are the raw GPS positions and the red dots are the corrected GPS positions with a Kalman filter. The moving speed is obtained by the displacement of the current position at *t* and previous position at *t* − 1, and it is assigned to a segmented IMU signal every 1 s (i.e., the GPS data update rate is 1 Hz) corresponding to the label directly.

Our proposed dataset collection scheme is shown in Figure 2. Based on the corrected and filtered pedestrian trajectory, we calculated the velocity using the displacement between the current time position and the previous time position at every measurement time, and we assigned the velocity to the label of the segmented IMU sensor signal frame. The displacement and moving speed can be computed using the following equation:

(1) $$\begin{array}{l} \Delta d_{t}=\sqrt{\;{\left(p_{t}-p_{t-1}\right)}^{2}\;},\;p_{t}=\left[\begin{array}{c} x_{t}\\ y_{t} \end{array}\right]\\ s_{t}=\frac{\Delta d_{t}}{t-(t-1)} \end{array},$$

where *p_t_* denotes the corrected GPS position *x, y* at time *t*, which are transformed into the longitude and latitude into the universal transverse mercator (UTM) coordinate system, respectively. Δ*d_t_* and *s_t_* are the displacement and the velocity computed using positions at time *t* and *t* − 1, respectively. In addition, *s_t_* is assigned to the label of the IMU sensor signal measured from *t* − 1 to *t*. The above process is repeated every second when walking outdoors where GPS is available, and the automatically generated dataset is used for learning.

### 3.2. Multiscale and Multiple 1D-CNN for Feature Extraction

The main difference between conventional CNNs and 1D-CNN is the use of a one-dimensional time-series signal as the input data instead of two-dimensional (2D) pixels or three-dimensional (3D) voxels. Multiple time-series data (i.e., IMU sensor data) is fed into the CNN as the input in our proposed systems. The key role of the CNN is to extract the correlation of spatially and temporally adjacent signals using nonlinear kernel filters. The local feature of the input data can be extracted by applying several kernel filters, and the global feature vector can be generated through several pairs of convolutional and pooling layers [26].

**Figure 3:**
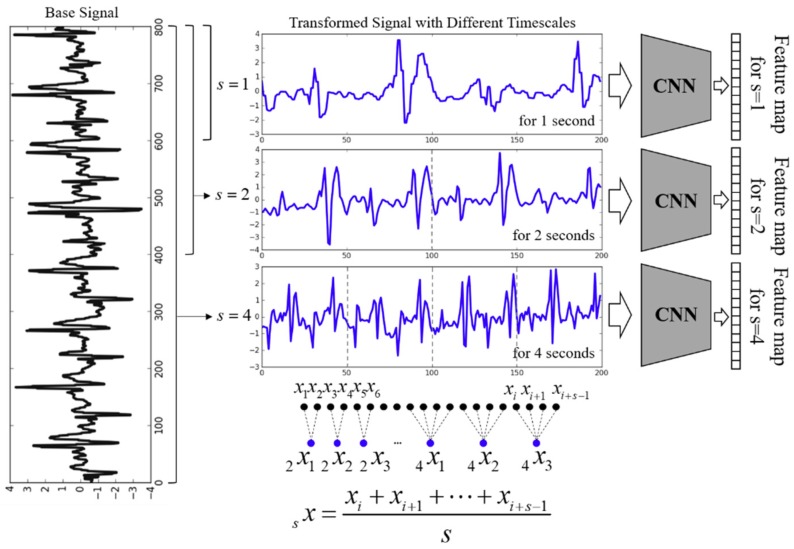
The proposed multiscale and multiple CNN scheme to extract features. Even if the scaling factor is different, the size of converted signals is same. The multi-scaled signals are fed into each CNN, and then each feature map is generated.

Our study focuses on indoor localization based on time-series sensor signal pattern learning under varying walking conditions. In such a case, a proper input data size considering the temporal properties should be considered to extract the features well. Therefore, we proposed a multiscale, multiple CNN architecture to incorporate inherent feature learning for various signal durations as well as overcome the limitations using only single input data in conventional CNN. This approach can simultaneously reflect the features of various input data measured for different time durations. The overall scheme of the multiscale and multiple CNN model to extract the feature is illustrated in Figure 3.

We motivated multiscale feature learning into the CNN architecture to improve the learning efficiency and extract more features [38]. However, the proposed multiscale method possesses the following two characteristics compared to conventional multiscale studies. First, the size of the converted signal by each scaling factor is always the same. Second, the feature vectors extracted by multiple CNNs are not combined into one feature map, and these feature vectors are independently used. The reason for our multiscale and multiple CNN concept is to utilize the same dimension as the input data for the RNN model, which will be introduced in Section 3.3.

Multiscale operation is performed by using the following expression:

(2) xjs=1s∑i=(j−1)s+1jsxi, 1≤j≤Ns,

where $x=\{x_{1},x_{2},\cdots ,x_{n}\}$ denotes a measured one-dimensional discrete time-series signal, *x_i_* is the sample value at time index *i*, *s* is a scaling factor, and *N* denotes the number of samples in each segmented signal frame. The time-series signal *x* is divided into a non-overlapped window length *s* (i.e., the scaling factor), and then, window *j*’s data points are consecutively averaged. We can construct a multi-scaled signal xs={x1s,⋯,xjs,⋯,xn/ss}. 

Typically, CNNs consist of several pairs of convolutional and pooling layers to extract the feature map, and learning generates the desired output by optimizing learnable parameters through feedforward and backpropagation passes [26]. The objective of using the CNN architecture in our study is to achieve feature extraction. The signals transformed by the multiscale operation are fed into multiple CNNs as the initial input data, and the feature map of multiple CNN based on the multiscaled signal can be expressed as:

(3) zjls=ReLU(∑l=1Ml−11dconv(xil−1s,kijl−1) + bjl),yjls=maxpooling(szjl),

where xil−1s and zjls denote the input and output of the convolutional layer with the ReLU = *max*(0, *x*) activation function, respectively; $k_{\mathrm{ij}}^{l-1}$ denotes the learnable kernel filter from the *i*th neuron in layer *l* − l to the *j*th neuron in layer *l*; and 1*dconv*() indicates the convolutional operation. $b_{j}^{l}$ is the bias of the *j*th neuron in layer *l*, and *M^l−^*^1^ denotes the number of kernel filters in layer *l* − 1. yils denotes the output of max pooling layer *l*, as well as the input of the next convolutional layer *l* + 1. Consequently, pairs of convolutional and pooling layers reconstruct a feature vector as the input of the above recurrent neural network model. Additional details of the backpropagation by minimizing the cost function are available in Lecun et al. [39].

Moreover, the multiscale operation can be considered as the moving average without an overlapped window for low-pass filtering. In other words, high-frequency components and random noises in a raw time-series sensory signal can be naturally filtered. Although CNNs have not been verified to eliminate noise well, lightweight preprocessing (such as smoothing and averaging) can naturally improve the performance [40].

### 3.3. Hierarchical Multiscale Recurrent Neural Networks

Our walking pattern learning model is mainly motivated by the following observation and considerations. According to studies on pedestrian movement characteristics [41,42] and our experimental experience, the stride and walking speed tend to remain stable as long as there is no change in the external environment or internal user behavior. In other words, within a relatively short period (i.e., in a few seconds), the previously estimated walking speed may affect the current working speed.

**Figure 4:**
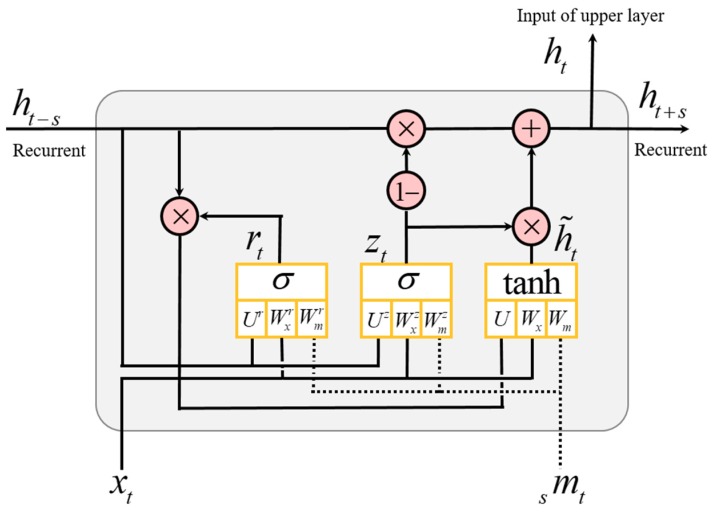
Our proposed enhanced gated recurrent unit (GRU) cell. The new components are displayed in dot-line. *m* is the feature vector of the multiscaled (*s*) signal at time *t*, and also, it is the same as the feature vector extracted from the last corresponding CNN layer by Equation (3). The output activation to the recurrent path is determined by the scaling factor *s*.

Learning the temporal representations and dependencies is one of the challenges of deep neural networks. Recurrent neural networks, such as LSTM and GRU, have been considered as promising methods to solve this problem [13]. Therefore, we designed a new scheme, which is referred to as a hierarchical multiscale recurrent neural network, that can capture temporal dependencies with different timescales using a novel update mechanism. Figure 4 presents our proposed enhanced GRU cell.

The GRU mainly consists of an update gate and a reset gate. The input of the GRU cell is *x_t_*, *_s_m_t_*, and *h_t_*_−*s*_, the output is *h_t_* and *h_t+s_* for time *t*. Both gates are similarly computed as in the following equation:

(4) update gate : zt=σ(Wxzxt+Uzht−s+Wmzmts+bz)reset gate : rt=σ(Wxrxt+Urht−s+Wmrmts+br),

where *W_x_*, *U_h_*, and *W_m_* denote the learnable parameters that linearly combine the input vector *x_t_*, previous hidden state output vector *h_t_*_−*s*_, and additional multiscaled input *_s_m_t_*, respectively, *b* is a bias, superscripts *z* and *r* mean that the corresponding parameter belongs to the update gate or the reset gate, and the activation function *σ* is a sigmoid. The difference of the basic GRU is that the scaling factor *s* determines whether the recurrent path is activated or not.

The candidate activation can be defined at a current state as follows:

(5) h˜t=tanh(Wxxt+U(rt∘ht−s)+Wmmts+bh),

where *r_t_* is a set of reset gates, ∘ is element-wise multiplication, and tanh is used as an activation function. The candidate activation $\tilde{h}$*_t_* is calculated by the current state *W_x_x_t_*, *W_ms_m_t_*, and previous hidden state *Uh_t_*_−*s*_, but it depends on the reset gate *r_t_* to activate the previous hidden state. The activation function of the reset gate is a sigmoid, *σ*(*x*) ∈ [0, 1]. If the reset gate value is 0, the previously computed state is forgotten, if it is 1, it allows it to maintain a previously computed state. The current state information is reflected, regardless of the reset gate.

The output *h_t_* of the GRU at time *t* is computed as follows:

(6) $$h_{t}=z_{t}h_{t-s}+\left(1-z_{t}\right)\circ {\tilde{h}}_{t},$$

where *h_t_*_−*s*_ is the previous activation and $\tilde{h_{t}}$ is the candidate activation of the current state, and the update gate *z_t_* determines how much it updates each component. The update gate also uses the sigmoid; if *z_t_* is 0, all of the previous activation is forgotten, and only $\tilde{h}$*_t_* is activated, if it is 1, the previous activation *h_t_*_−*s*_ is determined as the output of GRU.

**Figure 5:**
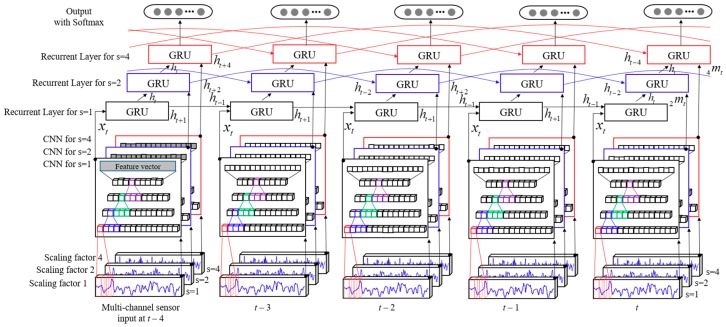
Our proposed hybrid multiscale convolutional and recurrent neural network model to train and estimate the moving speed for segmented and multiscaled sensory signal. The transformed signals with different timescales are fed into a corresponding CNN to extract the feature vector, and then, each feature vector is fed into the corresponding GRU cell as an additional input, *_s_m_t_*. Only the feature vector *x_t_* (*s* = 1) is fed into the first GRU layer of the stacked RNNs as the input, and *h_t_* is used for the input of the upper GRU layer. The recurrent activation at each GRU layer is determined by the scaling factor of the additional input *_s_m_t_*. Finally, the probability distribution for the target moving velocity is computed by Softmax.

Our proposed hybrid multiscale convolutional and recurrent neural network model to learn and classify the pedestrian walking velocity using a one-dimensional time-series signal extracted during walking with a smartphone is expressed in Figure 5.

## 4. Experimental Results

### 4.1. Experimental Setup

The dataset was automatically constructed while walking outdoors in the real world with smartphone sensors. We developed and utilized an Android application (app) that can measure and store the GPS and IMU sensors. The main scenario for our experiment is walking in a situation such as (i) *handheld*: hold the phone in front of the chest, (ii) *swing*: hold the phone in a swinging hand, and (iii) *pocket*: the phone in the trouser (back/front) pocket. We tried to walk as much as possible in real life when we collected data, and some data during calling, texting, and running were included in the dataset as well.

We used the tri-axial accelerometer in the IMU to measure the walking pattern with a 200-Hz sampling rate, and a magnetometer was not used due to the magnetic distortion caused by metal structures. We used the NMEA 0183 standard parser to get the GPS position, and transformed WGS84 to UTM coordination to compute the meter unit operation when a reliable fix of the GPS signal was obtained. The raw GPS position was corrected by a Kalman filter, and the displacement and velocity were calculated by Equation (1). Then, the velocity is assigned to the label of the measured sensory signal at the corresponding time. For the experiment, the tri-axial acceleration signals and the GPS position were sampled at 200 Hz and 1 Hz, respectively, and then they were stored in the Secure Digital (SD) memory card as log files. Therefore, we used a smartphone with an additional SD card slot. The log file consists of a list of measured data every 1 s (i.e., 200 × 3 × 4 bytes of acceleration data, 2 × 4 bytes of position data, 1 × 4 bytes of velocity data, the number 4 means a float type). For instance, approximately 8.7 MB of memory space is required to store the signal as a training dataset for 1 h.

The dataset was collected individually; it was composed of the measured IMU sensor and GPS data while walking 3.6 km to 41 km per subject, 3% to 11% of the collected data was eliminated from the dataset if the GPS reliability flag was not fixed. Naturally, the experiment was carried out using the user’s own walking pattern signal measured by their own smartphone.

We utilized Ubuntu 16.04LTS on a desktop, TensorFlow, and Nvidia GTX1060 for learning, and then we exported our trained deep learning model and learnable parameters (e.g., weights and biases) to an Android app. The training set and validation set were randomly allocated 80% and 20% of the entire dataset, respectively, and a test was performed indoors using the Android app implemented by us that can display the traveled distance in real-time. Running the app requires a total of 600 MB of memory space for the data file, which includes the weight parameters and the metafile, including the model structure. In addition, it takes an average of 310 ms on Galaxy S7 (higher than Android-Nougat) to convert the input signal into the output velocity when the other apps are not working.

As mentioned above, our proposed dataset-constructing method requests the users to generate data, because the dataset should only be composed of data based on our own walked signal, position, and velocity, and it is used to learn the user’s own walking patterns by the proposed deep learning model. This means that they need to run the specific app to collect data when they are outdoors. Although we manually stored the dataset on the server, it is also possible for users to send a collected dataset to a private cloud or private server using Wi-Fi or BLE. We managed each subject’s walking dataset on one desktop server, and generated a data file that contains the weighted parameters for each corresponding subject. Table 1 shows the structure and parameters of our proposed simplified hybrid CNN–RNN model.

### 4.2. Performance Evaluation

Before our proposed method was designed, we needed to confirm that the accuracy of the corrected GPS positioning was appropriate for labeling for the measured time-series sensory signal. Table 2 shows the displacement error between the actual displacement and the corrected GPS position by each distance along with a pre-defined straight path. In general, a commercial GPS has a positioning error of more than several meters in the global coordination system. According to our experiment, there was an average of 10.2 m of error in the global coordination in open space. However, we only needed to apply a relative coordination system to compute the displacement between the start and the end points. Thus, we determined that it is appropriate to estimate the moving speed of the pedestrian through a trajectory corrected within less than 2 m of average displacement error, and the error did not linearly increase with any increase in distance. We used a built-in microphone on the smartphone and a short whistle sound to check the start and end times. In order words, while measuring the GPS and IMU sensors, the microphone was also measured, and the whistle was used as a timing marker to compute the displacement error of the experimental result.

Four metrics were computed to quantify the performance of different models: *Accuracy* means a correctly classified sample from the total population, (*TP + TN*)/(*TP + TN + FP + FN*); *Precision* is the ratio of correctly predicted conditions to the total predicted positive conditions for each class, *TP/*(*TP + FP*); *Recall* presents the ratio of correctly predicted positive conditions to all of the true conditions for each class, *TP/*(*TP + FN*); and the *F* − 1 score refers to the weighted average of *Precision* and *Recall*, 2 × (*precision × recall*)/(*precision + recall*). The definitions of the above metrics use the true positive (*TP*), true negative (*TN*), false positive (*FP*), and false negative (*FN*). Another performance metric, the distance error, means the difference the between actual and predicted traveled distance over the whole validation set.

Table 3 presents the performance of our proposed hybrid multiscale convolutional and recurrent neural network model with a mean of fivefold cross-validation, and the results are also compared to those of other models. The training and validation set were randomly allocated 80% and 20% data, respectively. In other models except for the proposed model, the segmented 2-s signal frame was fed into each model as the input. In this experiment, we used a dataset that walked around 41 km for subject 1.

As expected before the experiment, the performance outperformed when using CNN than when using ANN without any manually-designed features and a basic RNN model, which means that the appropriate features are automatically extracted through a CNN learning process. Even though CNN’s automatic feature extraction does not always outperform a handcrafted feature extraction scheme, it can drastically reduce the time and cost for the feature analysis. The performance of the LSTM and GRU, which can solve the long-term dependency, improved more than that for the CNN without considering temporal correlation and basic RNN models. According to our experimental result and other studies, both models show enhanced performance, the performance between LSTM and GRU was insignificant, hyperparameter tuning seems to be more important than the model chosen. Thus, we adapted GRU as a part of our proposed model, considering that it has a small number of parameters and short training time compared to the LSTM. Hybrid CNN and GRU were performed to reflect the effect of the CNN’s automatic feature extraction, and it showed a slight improvement in the performance. Our proposed model demonstrated the best performance, we reduced a traveled distance prediction error to 1.27% with simple noise rejection and a trend of a different time scale using the multiscale method, enhanced GRU cell, and novel hybrid model architecture.

In addition, we compared the classification and the regression methods that have different ways of predicting the output. These two methods exhibit a difference in the dataset configuration. When the case of the regression method is applied, the label for the corresponding signal in the dataset is directly assigned using the calculated velocity from the corrected GPS trajectory. In the other case, the calculated velocities were clustered using an expectation-maximization (EM) algorithm, and then the representative values for each cluster were assigned to the label. The performance of the regression methods was slightly lower, even though a calibrated trajectory was used, and it seems that slightly different velocities are assigned as labels to a similar signal pattern.

**Figure 6:**
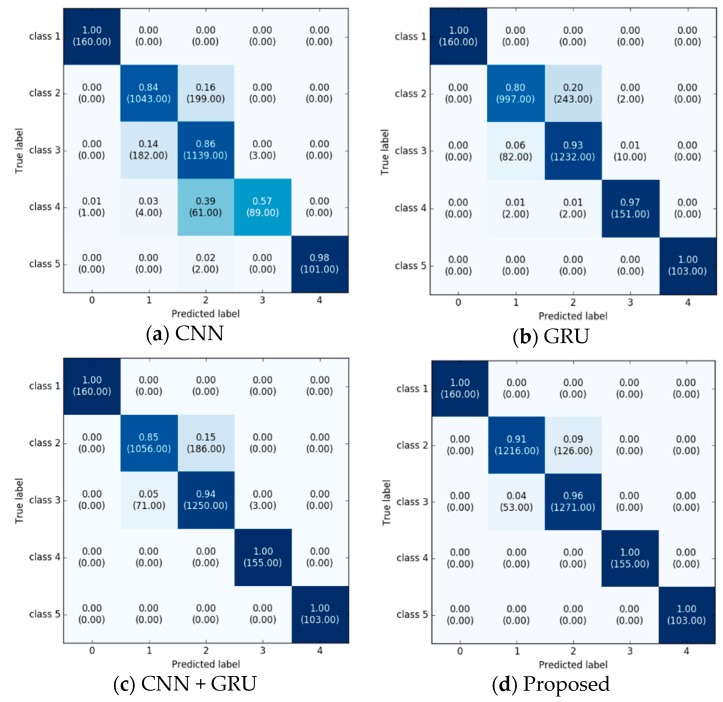
Confusion matrix with normalization for classification results of Table 3. The number of samples predicted for corresponding class is presented in the parentheses. (**a**) Represents the result of CNN; (**b**) shows the result of GRU; (**c**) is the result of basic hybrid CNN and GRU model; (**d**) represents the result of our proposed model.

The confusion matrix for the classification results is shown in Figure 6 for comparison with other methods. Classes 1 and 5 are the stationary and fastest class, respectively, so these are relatively well classified in all of the models. This means that these two classes are distinct compared to the other class signal patterns. Applying the enhanced deep learning model, the classification accuracy for classes 2 and 3 gradually improved.

We exported and modified the trained proposed deep learning model to an Android app using TensorFlow Mobile to carry out the experiment indoors. The app works online and displays the traveled distance for the measured signal in real-time. We carried out an experiment with nine subjects to verify that the method of estimating the travel distance after learning their own walking pattern via their own smartphone outperforms previously studied smartphone-based PDR methods that use the step counts and step length estimation.

The experiments were carried out in a 60-m indoor corridor; subjects 1 to 6 walked with varying speeds every 20 m, and subjects 7 to 9 walked at the constant speed. Table 4 shows the duration and distance of the walking dataset collected by each subject, and corresponding smartphone devices for training. The experimental results of our proposed model and the existing methods are shown in Table 5. The proposed method that learns the user’s walking pattern using their own smartphone showed the best performance, because it was considered by any change in the device, moving speed, and walking pattern, as well as the smartphone’s orientation and placement. Since the proposed method is based on deep learning, the accuracy tends to improve as the amount of data increases. According to the experimental results, when using more than 10 km of a walking dataset, less than 2.4% and up to 1.5% of a distance error is generated.

In the experiment based on Weinberg [12], although the *K* parameter was tuned for each subject, the performance deviation was large due to the change in the orientation and position of the smartphone according to the walking pattern, as well as the variation in the walking speed. Ho et al. [11] suggested a way to control the *K* parameter of Weinberg et al. [12] according to the walking speed change. However, this did not solve the problem caused by the unconstrained walking pattern either. Huang et al. [23] proposed a method to estimate the stride length when only the smartphone was held in a swinging hand by exploiting the cyclic features of walking, in order to overcome the limitation of carrying a smartphone in front of the chest in most smartphone-based PDR approaches. However, manually designed cyclic features based on a biomechanical model cannot reflect the diversity of each pedestrian’s swinging action as well as the variation in the walking pattern; thus, a large performance deviation among the subjects is shown in the result.

In addition, to verify the performance compared with a deep learning-based model, we performed the experiment using the ANN-based model [35] that uses five parameters as the input closely related to the stride length and one output to estimate the corresponding stride length. For this experiment, we reconfigured the existing dataset with a pair of five parameters (e.g., stride frequency, maximum, standard deviation, mean of acceleration, and height of subject) and stride length, and these parameters and stride length were extracted from the measured IMU sensory signal and corrected GPS trajectory, respectively. Although we were not sure that the ANN’s model size, hyperparameters, and optimization techniques were designed well to achieve the best performance, we confirmed that the proposed multiscale hybrid CNN–RNN model considering nonlinear feature vectors from multiple time scale signals and temporal correlation can be more effective to learn multivariate time-series signal patterns.

## 5. Discussion and Conclusions

Stride length estimation is one of the most important factors in PDR-based indoor localization, and it is affected by the pedestrian’s movement behavior, speed, and physical properties as well as the smartphone’s placement and orientation. Under the conditions mentioned above, precise estimation of the stride length is a challenging research topic. Although many studies have used network infrastructure such as Wi-Fi and BLE to calibrate and overcome the drawbacks of PDR, it is limited only to infrastructure that is based indoors. Therefore, we introduced a new precise PDR approach that can be utilized without network infrastructure by learning the personal walking pattern during the various activities in everyday life.

Since the dataset used in our experiment is automatically constructed, it may be of lower quality than a dataset measured under controlled experimental conditions. However, the experimental results showed an approximately 2% distance error when the data was collected over 10 km, and an approximately 1% error when the data was collected over 30 km. Although our experiments were carried out under limited conditions (i.e., three walking types) to apply the same condition to the subjects, we expect to outperform these if a larger dataset (e.g., longer period of time, more variable walking pattern, and smartphone placements) is conducted and the accuracy of the data collection has improved.

In addition, although this paper focuses on solving the problems of indoor localization applications, a multiscale hybrid convolutional and recurrent neural network model can provide a general time-series signal classification, regression, and forecasting framework that accommodates a wide range of applications i.e., collected signals via other single or multiple sensors that can be applied in our designed model directly. However, there is one thing that needs to be improved; at present, we need to learn the entire dataset when additional data with a new output class (i.e., velocity) is generated, because our proposed learning scheme uses a conventional supervised learning approach.

In the future, we would need to focus on researching how to set the initial position using minimum reference points, how to estimate the direction based on the proposed model, and how to match and correct the final position to the map during walking indoors to solve the cumulative error of a wrong estimation in the moving speed by using the minimum reference points, such as beacons and landmarks (e.g., corners or objects). Moreover, we will study online and lifelong [43] learning to overcome the inherent limitations of supervised learning, such as fixed output classes and additional datasets.

Finally, we hope that a major contribution of our study will be to motivate researchers who want to apply a new and easy approach for indoor localization as well as time-series sensory signal analysis.

## Figures and Tables

**Table 1:** Simplified Hybrid Single-Scale CNN–Gated Recurrent Unit (GRU) Model Structure and Parameters.

Structure	Input (I)	Filter	Depth	Stride	Output (O)	Number of Parameters
Conv1 + Relu	200 × 1 × 3	4 × 1	64	1	197 × 1 × 64	(4 × 1 × 3 + 1) × 64 = 832
Max Pooling (dropout 0.2)	197 × 1 × 64	2 × 1			98 × 1 × 64	
Conv2 + Relu	98 × 1 × 64	4 × 1	64	1	95 × 1 × 64	(4 × 1 × 64 + 1) × 64 = 16,448
Max Pooling (dropout 0.2)	95 × 1 × 64	2 × 1			47 × 1 × 64	
Conv3 + Relu	47 × 1 × 64	4 × 1	64	1	44 × 1 × 64	(4 × 1 × 64 + 1) × 64 = 16,448
GRU	44 × 1 × 64				128	2 × 3(I^2^ + I × O + I) = 49,758,720
Output Classes	128				5	128 × 5 = 640
Overall						49,793,088

**Table 2:** Displacement Error of Corrected GPS Trajectory in Comparison with Ground Truth.

Type	Distance (m)/Mean ± Std				
	25 m	50 m	75 m	100 m	Average
Handheld	1.53 ± 0.45	0.83 ± 0.27	1.29 ± 0.22	2.44 ± 0.56	1.52
Swing	2.55 ± 0.49	2.25 ± 0.20	1.23 ± 0.28	1.94 ± 0.32	1.99
Pocket	2.28 ± 0.82	2.88 ± 0.49	1.64 ± 0.68	2.05 ± 0.88	1.96
Mix	2.57 ± 0.55	0.87 ± 0.26	1.26 ± 0.48	1.61 ± 0.45	1.58
Average	1.79 m	1.28 m	1.50 m	2.08 m	1.66 m

**Table 3:** Signal Classification Performance of the Proposed Model In Comparison with Other Models.

Models	Evaluation Parameters				
	Distance Error (%)	Accuracy	Precision	Recall	F − 1 Score
ANN	6.466	0.668	0.564	0.536	0.538
CNN	3.599	0.878	0.852	0.848	0.87
Vanilla RNN	5.676	0.714	0.643	0.633	0.629
LSTM	2.441	0.904	0.895	0.888	0.887
GRU	2.379	0.903	0.890	0.885	0.885
CNN + GRU	1.860	0.925	0.915	0.913	0.912
Multiscale CNN + GRU with classification (Proposed)	1.278	0.949	0.943	0.942	0.942
Multiscale CNN + GRU with regression	1.572	-			

**Table 4:** Properties and Walking Dataset for Each Subject.

Types	Subject 1	Subject 2	Subject 3	Subject 4	Subject 5	Subject 6	Subject 7	Subject 8	Subject 9
Smartphone	Samsung Galaxy Note 7	Samsung Galaxy S8	LG V30	Samsung Galaxy S7	LG G6	LG V30	Samsung Galaxy Note 7	Samsung Galaxy S8+	Samsung Galaxy S7
Dataset configuration for training	14.4 h, 41 km	11.1 h, 34 km	10.1 h, 31 km	4.0 h, 22 km	4.9 h, 16 km	4.3 h, 14 km	2.2 h, 10 km	2.4 h, 7 km	1.3 h, 3.6 km
Pedestrian properties	181 cm, 85 kg, 38 age, male	175 cm, 68 kg, 30 age, male	179 cm, 78 kg 30 age, male	173 cm, 75 kg, 28 age, male	163 cm, 53 kg, 24 age, female	177 cm, 79 kg, 35 age, male	172 cm, 70 kg, 27 age, male	160 cm, 55 kg, 21 age, female	161 cm, 48 kg, 28 age, female

**Table 5:** Evaluation and Analysis of Traveled Distance Estimation for Indoor Localization.

Models		Subject 1	Subject 2	Subject 3	Subject 4	Subject 5	Subject 6	Subject 7	Subject 8	Subject 9	Average
Proposed Method	Handheld	1.12 ± 0.14	1.01 ± 0.34	1.66 ± 0.49	2.02 ± 0.33	**0.89** ± 0.07	1.51 ± 0.17	1.42 ± 0.14	1.98 ± 0.38	3.02 ± 0.75	**1.63 m**
	Swing	1.05 ± 0.27	1.16 ± 0.29	**1.03** ± 0.15	1.99 ± 0.24	1.12 ± 0.37	1.60 ± 0.40	1.45 ± 0.61	2.63 ± 0.63	3.55 ± 0.64	**1.74 m**
	Pocket	0.78 ± 0.07	**0.65** ± 0.15	0.87 ± 0.21	1.18 ± 0.19	1.04 ± 0.21	2.37 ± 0.27	1.40 ± 0.41	2.73 ± 0.35	3.42 ± 0.67	**1.60 m**
Weinberg [12]	Handheld	12.7 ± 0.74	13.8 ± 0.29	8.12 ± 1.15	12.4 ± 0.67	12.3 ± 1.36	8.73 ± 1.57	4.27 ± 2.71	**3.67** ± 0.86	3.95 ± 0.97	**8.90 m**
	Swing	25.26 ± 3.78	22.16 ± 4.14	29.58 ± 2.70	27.05 ± 2.01	20.46 ± 2.16	25.12 ± 2.21	20.88 ± 5.39	17.44 ± 3.12	**16.64** ± 3.17	**22.73 m**
	Pocket	16.20 ± 0.95	18.23 ± 2.09	20.30 ± 1.94	17.02 ± 2.21	25.91 ± 3.29	18.18 ± 4.92	13.75 ± 2.70	**12.37** ± 0.94	14.20 ± 1.58	**17.35 m**
Ho et al. [11]	Handheld	6.30 ± 3.82	3.01 ± 3.81	4.49 ± 3.00	3.48 ± 1.61	3.18 ± 1.88	3.46 ± 1.22	**2.43** ± 2.55	6.39 ± 3.46	4.93 ± 4.25	**4.19 m**
	Swing	**13.73** ± 2.66	18.26 ± 3.40	21.65 ± 3.84	17.65 ± 3.61	20.26 ± 2.62	22.14 ± 1.79	18.67 ± 3.22	20.57 ± 3.71	16.39 ± 2.58	**18.81 m**
	Pocket	17.37 ± 3.89	12.63 ± 5.15	14.11 ± 1.48	15.13 ± 2.19	16.35 ± 2.18	15.72 ± 5.74	10.82 ± 1.07	13.63 ± 2.22	**10.33** ± 2.26	**14.01 m**
Huang et al. [23]	Handheld	-	-	-	-	-	-	-	-	-	-
	Swing	**3.24** ± 1.10	4.22 ± 2.27	11.39 ± 1.90	3.38 ± 3.00	16.03 ± 6.14	15.31 ± 6.14	7.25 ± 4.23	12.21 ± 3.90	4.24 ± 1.81	**8.58 m**
	Pocket	-	-	-	-	-	-	-	-	-	-
Xing et al. [35]	Handheld	**2.50** ± 0.52	2.75 ± 0.26	3.19 ± 0.70	3.61 ± 1.68	4.28 ± 0.97	5.93 ± 0.40	3.98 ± 2.13	5.53 ± 0.94	5.76 ± 1.06	**4.17 m**
	Swing	4.17 ± 0.95	**3.94** ± 1.92	5.93 ± 0.38	4.08 ± 0.41	5.69 ± 2.17	6.13 ± 0.68	7.54 ± 0.81	6.57 ± 1.00	7.48 ± 0.66	**5.73 m**
	Pocket	5.21 ± 0.98	**4.94** ± 0.97	7.64 ± 1.11	6.42 ± 1.76	7.14 ± 1.15	8.02 ± 1.62	8.83 ± 0.43	9.29 ± 2.52	8.28 ± 1.12	**7.31 m**

## References

[1] Brena R.F., García-Vázquez J.P., Galván-Tejada C.E., Muñoz-Rodriguez D., Vargas-Rosales C., Fangmeyer J. (2017). Evolution of Indoor Positioning Technologies: A Survey. J. Sens..

[2] Pavel D., Robert P. (2017). A survey of selected indoor positioning methods for smartphones. IEEE Common. Surv. Tutor..

[3] Hui L., Houshang D., Pat B., Jing L. (2007). Survey of wireless indoor positioning techniques and systems. IEEE Trans. Syst. Man Cybern..

[4] Xiaohua T., Ruofei S., Duowen L., Yutian W., Xinbing W. (2017). Performance analysis of RSS fingerprinting based indoor localization. IEEE Trans. Mobile Comput..

[5] Luo H., Zhao F., Jiang M., Ma H., Zhang Y. (2017). Constructing an indoor floor plan using crowdsourcing based on magnetic fingerprinting. Sensors.

[6] Sabet M.T., Daniali H.R.M., Fathi A.R., Alizadeh E. (2017). Experimental analysis of a low-cost dead reckoning navigation system for a land vehicle using a robust AHRS. Robot. Auton. Syst..

[7] Kang W., Han Y. (2015). SmartPDR: Smartphone-based pedestrian dead reckoning for indoor localization. IEEE Sens. J..

[8] Zhang W., Li X., Wei D., Ji X., Yuan H. A Foot-Mounted PDR System Based on IMU/EKF + HMM + ZUPT + ZARU + HDR + Compass Algorithm. Proceedings of the 2017 International Conference on Indoor Positioning and Indoor Navigation (IPIN).

[9] Yu N., Zhan X., Zhao S., Wu Y., Feng R. (2018). A precise dead reckoning algorithm based on bluetooth and multiple sensors. IEEE Internet Things J..

[10] Kang X., Huang B., Qi G. (2018). A novel walking detection and step counting algorithm using unconstrained smartphones. Sensors.

[11] Ho N.-H., Truong P.H., Jeong G.-M. (2016). Step-detection and adaptive step-length estimation for pedestrian dead-reckoning at various walking speeds using a smartphone. Sensors.

[12] Weinberg H. (2002). Using the ADXL202 in Pedometer and Personal Navigation Application.

[13] Chung J., Gulcehre C., Cho K., Bengio Y. Empirical evaluation of gated recurrent neural networks on sequence modeling. Proceedings of the Deep Learning and Representation Learning Workshop: NIPS.

[14] Alarifi A., Al-Salman A., Alsaleh M., Alnafessah A., Al-Hadhrami S., Al-Ammar M.A., Al-Khalifa H.S. (2016). Ultra wideband indoor positioning technologies: Analysis and recent advances. Sensors.

[15] Wang X., Gao L., Man S., Pandey S. (2017). CSI-based fingerprinting for indoor localization: A deep learning approach. IEEE Trans. Veh. Technol..

[16] Li J., Guo M., Li S. Smartphone-Based Indoor Localization with Bluetooth Low Energy Beacons. Proceedings of the 2nd International Conference on Frontieres of Science and Technology (ICFST-18).

[17] Luo J., Fu L. (2017). A smartphone indoor localization algorithm based on WLAN location fingerprinting with feature extraction and clustering. Sensors.

[18] Shu Y., Bo C., Shen G., Zhao C., Li L., Zhao F. (2015). Magicol: Indoor localization using pervasive magnetic field and opportunistic WiFi sensing. IEEE J. Sel. Areas Commun..

[19] Xie H., Gu T., Tao X., Ye H., Lu J. (2016). A reliablility-augmented particle filter for magnetic fingerprinting based indoor localization on smartphone. IEEE Trans. Mobile Comput..

[20] Yang Z., Wu C., Zhou Z., Zhang X., Wang X., Liu Y. (2015). Mobility increases localizability: A survey on wireless indoor localization using inertial sensors. ACM Comput. Surv..

[21] Wu C., Yang Z., Liu Y., Xi W. (2013). WILL: Wireless indoor localization without site survey. IEEE Trans. Parallel Distrib. Syst..

[22] Goyal P., Ribeiro V.J., Saran H., Kumar A. Strap-down Pedestrian Dead-Reckoning System. Proceedings of the International Conference on Indoor Positioning and Indoor Navigation.

[23] Huang B., Qi G., Yang X., Zhao L., Zou H. Exploiting cyclic features of walking for pedestrian dead reckoning with unconstrained smartphones. Proceedings of the 2016 ACM International Joint Conference on Pervasive and Ubiquitous Computing.

[24] Kang W., Nam S., Han Y., Lee S. Improved Heading Estimation for Smartphone-Based Indoor Positioning Systems. Proceedings of the 2012 IEEE 23rd International Symposium on Personal, Indoor and Mobile Radio Communications—(PIMRC).

[25] Bao H., Wong W.-C. A Indoor Dead-Reckoning Algorithm with Map Matching. Proceedings of the 9th International Wireless Communications and Mobile Computing Conference (IWCMC).

[26] Schmidhuber J. (2015). Deep learning in neural networks: An overview. Neural Netw..

[27] Liu W., Wang Z., Liu X., Zeng N., Liu Y., Alsaadi F.E. (2017). A survey of deep neural network architectures and their application. Neurocomputing.

[28] Kiranyaz S., Ince T., Gabbouj M. (2015). Real-time patient-specific ECG classification by 1D convolutional neural networks. IEEE Trans. Biomed. Eng..

[29] Ince T., Kiranyaz S., Eren L., Askar M., Gabbouj M. (2016). Real-time motor fault detection by 1-D convolutional neural networks. IEEE Trans. Ind. Electron..

[30] Abdeljaber O., Avci O., Kiranyaz S., Gabbouj M., Inman D.J. (2017). Real-time vibration-based structural damage detection using one-dimensional convolutional neural networks. J. Sound Vib..

[31] Ha S., Choi S. Convolutional Neural Networks for Human Activity Recognition Using Multiple Accelerometer and Gyroscope Sensors. Proceedings of the International Joint Conference on Neural Networks (IJCNN).

[32] Ordonez F.J., Roggen D. (2016). Deep convolutional and LSTM recurrent neural network for multimodal wearable activity recognition. Sensors.

[33] Yao S., Hu S., Zhao Y., Zhang A., Abdelzaher T. Deepsense: A Unified Deep Learning Framework for Time-Series Mobile Sensing Data Processing. Proceedings of the 26th International Conference on World Wide Web.

[34] Edel M., Koppe E. An Advanced Method for Pedestrian Dead Reckoning Using Blstm-Rnns. Proceedings of the International Conference on Indoor Positioning and Indoor Navigation (IPIN).

[35] Xing H., Li J., Hou B., Zhang Y., Guo M. (2017). Pedestrian Stride Length Estimation from IMU measurements and ANN based algorithm. J. Sens..

[36] Hannink J., Kautz T., Pasluosta C.F., Barth J., Schulein S., GaBmann K.G., Klucken J., Eskofier B.M. (2018). Mobile stride length estimation with deep convolutional neural network. IEEE J. Biomed. Health Inf..

[37] Hannink J., Kautz T., Pasuosta C.F., Gabmann K.-G., Kluchen J., Eskofier B.M. (2017). Sensor-based gait parameter extraction with deep convolutional network. IEEE J. Biomed. Health Inf..

[38] Cui Z., Chen W., Chen Y. (2016). Multi-scale convolutional neural networks for time series classification. arXiv.

[39] Lecun Y., Bottou L., Orr G.B., Muller K.-R. (2012). Efficient backprop. Neural Networks: Tricks of the Trade LNCS.

[40] Kang J., Park Y.-J., Lee J., Wang S.-H., Eom D.-S. (2018). Novel leakage detection by ensemble CNN-SVM and graph-based localization in water distribution systems. IEEE Trans. Ind. Electron..

[41] Hak L., Houdijk H., Beek P.J., Dieen J.H.V. (2013). Steps to take to enhance gait stability: The effect of stride frequency, stride length, and walking speed on local dynamic stability and margins of stability. PLoS ONE.

[42] Sutherland D. (1997). The development of mature gait. Gait Posture.

[43] Parisi G.I., Tani J., Weber C., Wermter S. (2017). Lifelong learning of human actions with deep neural network self-organization. Neural Netw..

